# Evaluation of Energy Utilisation Efficiencies of Digestible Macronutrients in Juvenile Malabar Snapper (*Lutjanus malabaricus*) Reveals High Protein Requirement for Optimal Growth Using Both Factorial and Multifactorial Approaches

**DOI:** 10.1155/anu/5467206

**Published:** 2024-12-21

**Authors:** Si Yan Ngoh, Xueyan Shen, Leo Nankervis, Katheline Hua

**Affiliations:** ^1^Tropical Future Institute, James Cook University, Singapore, Singapore; ^2^Marine Aquaculture Centre, Aquaculture Department, Urban Food Solution Division, Singapore Food Agency, Singapore, Singapore; ^3^Centre for Sustainable Tropical Fisheries and Aquaculture, James Cook University, Townsville, Australia

**Keywords:** digestibility, energy utilisation, growth, lipogenesis, *Lutjanus malabaricus*, macronutrients

## Abstract

Malabar snapper (*Lutjanus malabaricus*) is an economically important marine fish throughout the Indo-Pacific, with an emerging aquaculture industry. Although generic marine feeds are available for production, these are not optimised for this species. Understanding energy utilisation and balance can provide insight into suitable macronutrient profiles for new species to provide a baseline for future development. This study, therefore, evaluated the effect of dietary macronutrient composition (i.e., protein, fat, and carbohydrate) on the utilisation efficiencies of digestible energy (DE) in juvenile Malabar snapper using two isonitrogenous diets (high fat: HF and low fat: LF) with contrasting fat and carbohydrate content. Each diet was fed at four feeding levels (100%, 75%, 50%, and 25% apparent satiation) for 56 days, creating a 2 by 4 factorial design. The maintenance energy requirement of Malabar snapper was estimated to be 76.7 kJ kg^−0.8^ day^−1^, while the utilisation efficiencies of digestible protein (DP) and fat were 73.6% and 68.3%, respectively. Fish fed with LF, which has lower dietary fat and higher dietary carbohydrate levels, had significantly reduced energy utilisation efficiency for growth and significantly higher partial energy utilisation efficiency of digestible fat (DF) (*p*  < 0.05). Since body moisture is usually proportional to body fat content in fish, this implies that the energy from carbohydrates preferentially enters lipogenesis rather than being available for somatic growth, and adiposity does not directly result in weight gain. Malabar snapper utilises DF in preference to protein for metabolism, demonstrating a protein-sparing effect from lipids at DE intake levels below the maintenance requirement. Conversely, given the higher efficiency of fat retention than protein retention, protein is likely used before fat when energy intake is above maintenance. These findings suggest that Malabar snapper requires high levels of DP in its diet to support growth and that energy from dietary carbohydrates is diverted towards adiposity, consequently reducing growth.

## 1. Introduction

Macronutrients (i.e., protein, fat, and carbohydrate) from feed and feedstuffs are sources of energy intake essential for fish growth, reproduction, and health [1]. Dietary energy intake available for growth has been measured using a variety of energy evaluation strategies based on either digestible energy (DE), metabolisable energy (ME), or net energy (NE) approaches [2]. The common method to estimate the maintenance energy requirement, energy utilisation efficiency, and energy partitioning of protein and fat synthesis by a factorial approach (i.e., a factorial design and regression analysis approach) based on either DE or ME has been performed on several fish species. These species include gilthead seabream (*Sparus aurata*), European seabass (*Dicentrarchus labrax*) [3], Atlantic salmon (*Salmo salar*) [4], rainbow trout (*Oncorhynchus mykiss*) [5], and barramundi (aka Asian seabass, *Lates calcarifer*) [6]. The limitations of the factorial approach have been discussed by Milgen and Noblet [7]. Briefly, the DE or ME utilisation efficiency for growth (*k*_gDE_ or *k*_gME_) is estimated by the regression slope of retained energy (RE) on DE or ME intake and has been considered consistent within a fish species. In other words, energy utilisation efficiency has been assumed to be independent of the macronutrient composition in feed. However, many studies have reported that the *k*_gDE_ differs with different feed formulations in fish species [6, 8–11]. These discrepancies in *k*_gDE_ arose from variable feedstuffs used in feed formulations, demonstrating the influence of dietary macronutrient compositions on DE utilisation and highlighting the need to account for energy utilisation efficiency based on digestible macronutrients.

A multifactorial approach (i.e., a factorial design and multiple regression analysis approach) initially applied in swine studies [12] to assess the energy utilisation efficiency of individual macronutrients based on NE has been adapted for fish. Six fish species were investigated: Nile tilapia (*Oreochromis niloticus*), rainbow trout [13], common carp (*Cyprinus carpio*), barramundi [14], African catfish (*Clarias gariepinus*) [11] and snakehead (*Channa striata*) [10]. Results revealed that among these species, the energy utilisation efficiencies of digestible protein (*k*_NE;DP_) ranged from 47% to 86%, while those of digestible fat (*k*_NE;DF_) were between 86% and 95%. A comparison between carnivorous fish (i.e., snakehead, barramundi, and rainbow trout) and omnivorous species (i.e., Nile tilapia, African catfish, and common carp) indicated that carnivorous fish exhibited lower energy utilisation efficiencies of carbohydrates (*k*_NE;DC_) for growth, between 5% and 18%, and are unable to utilise digested carbohydrate efficiently compared to omnivorous fish, for which *k*_NE;DC_ ranged from 59% to 66%. Due to the poor utilisation of dietary carbohydrates by carnivorous fish, dietary protein and fats are the primary sources of dietary energy for growth and metabolism [1]. In the absence of sufficient dietary fats or carbohydrates to meet energy demands, a portion of digested protein must be diverted to support basal metabolism, resulting in lower protein utilisation efficiency for growth. By contrast, adequate supplementation of dietary fats to meet metabolic requirements enables fish to optimise DP intake for growth, a phenomenon referred to as the protein-sparing effect of lipids [15, 16].

Malabar snapper (*Lutjanus malabaricus*) is an important commercial fish commonly found in the tropical and subtropical waters of the Indo-Pacific region [17–19]. In Singapore, there is growing interest in farming Malabar snapper due to its high market value and consumer acceptance. However, to our knowledge, no specific feeds are available that are tailored to the nutritional needs of this species. Formulation of nutritionally optimised diets requires the understanding of nutrient requirements and utilisation efficiencies of the target species, but available information regarding the nutrient requirements of snappers (*Lutjanus* spp.) is limited and variable, and the nutritional requirements for Malabar snapper have not been studied. Snappers are carnivorous fish that primarily prey on crustaceans, cephalopods, and other benthic invertebrates [20]. Currently, their dietary protein requirements range between 32% and 45% [21–25], with recommended dietary fat content between 6% and 10% [23, 26]. Additionally, the optimal protein-to-energy ratio for juvenile mangrove red snapper ranges from 20.1 to 23.3 g MJ^−1^ [21, 25, 27]. While these nutritional studies on other snapper species provide valuable insights for formulating experimental feeds for Malabar snapper, no research has studied the *k*_gDE_ in any snapper species.

Notably, previous studies on other fish species have utilised diets with variable macronutrient compositions and energy intake to study the energy demands and utilisation efficiencies for growth [10, 11, 13, 14]. Those studies have not shown whether the *k*_gDE_ could be affected by differences in dietary fat or carbohydrate composition under similar DP intake and possibly demonstrate any protein-sparing effect from fat or carbohydrate. Therefore, we propose to investigate the *k*_gDE_ using two isonitrogenous diets with varying fat and carbohydrate content and four feeding levels to create a significant contrast in DE intake. Given the absence of prior studies on the nutritional profile of Malabar Snapper, this study aims to elucidate macronutrient utilisation and deposition patterns for this species. Specifically, we aim to determine the availability of energy from DP, DF, and DC and to determine their utilisation efficiency for growth and energy deposition.

## 2. Materials and Methods

### 2.1. Experimental Diets

Two isonitrogenous diets, HF (high-fat, low-carbohydrate) and LF (low-fat, high-carbohydrate), were designed with different proportions of fish meal (HF: 45% vs. LF: 30%) and contrasting fat and carbohydrate content (Table 1). Both diets were formulated to meet or exceed the known nutritional requirements of other snapper species. On a dry matter (DM) basis, the fat content of HF was 15.2% compared to LF at 6.6%, and the carbohydrate content of HF was 23.6% compared to LF at 31.7%. The crude protein (CP) content of LF was formulated to resemble the HF by replacing fish meal with plant-based proteins such as SBM, SPC, and CGM. The contrasting fat and carbohydrate contents were formulated by differing the proportions of wheat flour and fish oil, while the corn starch content in both diets was constant. Detailed information on the amino acid requirements of snapper is scarce. To prevent deficiency in essential amino acids, the diets were formulated to meet the known amino acid requirements of marine fish [28].

Both HF and LF were manufactured in the pilot feed mill at the Marine Aquaculture Centre, Singapore (MAC). Ingredients were milled and sieved to prevent large particles (>0.5 mm) from clogging the extrusion process. The ingredients, except fish oil, were mixed homogeneously with 1 g kg^−1^ of Yttrium (III) oxide added as an inert digestion marker for 30 min using a 100 L dry powder horizontal paddle mixer (KSE-PM100, Kong Shiang Engineering Pte. Ltd.) before extrusion. The experimental diets were extruded using a co-rotational twin-screw extruder (Evolum 25, Clextral) using a 3.5 mm die insert and with the temperature set at 100°C at the barrel end. Post-extrudate pellets were dried below 10% moisture level using a fluidised bed-dryer at 70°C for at least 30 min. Dust and small particles were removed by sieving before fish oil was vacuum coated at 200 mbar of absolute pressure. The experimental diets were left to cool at ambient temperature and stored at 4°C before use.

### 2.2. Feeding Trial

Juvenile Malabar snapper (*L. malabaricus*) were obtained from MAC, and the experiment was conducted in MAC's shared experimental tank system, which comprised 36 replicated 250 L fibreglass rectangular tanks with the sloped bottom (size: 0.27 m^3^, length: 0.83 m, width: 0.52 m, and depth: 0.68 m). Each of the tanks is fitted with an individual faecal sedimentation column for the collection of faecal matter for apparent digestibility estimation. Before the start of the experiment, fish were acclimatised to the system for 1 week and fed to apparent satiation with a commercial diet (M503, Uni-president Vietnam Co. Ltd., Vietnam). At the start of the experiment, 960 fish were selected through manual grading by size. Batches of 30 fish with an average initial body weight (BW) of 57.84 g (SD 0.13) were weighed and randomly allocated to one of the 32 fibreglass tanks; four remaining tanks in the system were unused. The treatment groups are conducted in quadruplicate (*n* = 4), with tank configuration randomised using the sample function in *R* [29]. The experiment was conducted under flow-through seawater conditions with a water exchange rate of 150% per hour. Throughout the experiment, the daily outlet water parameters were monitored using Aqua Troll 500 (In-Situ, Colorado). The mean water temperature recorded was 29.6°C (SD 0.38), the mean dissolved oxygen level was 6.34 mg/L (SD 0.19), and the mean pH was 8.3 (SD 0.07). Ammonia, nitrite, and nitrate concentrations in rearing water were 0 mg/L (API saltwater master test kit). A photoperiod of 12 h of light and 12 h of dark was maintained throughout the experiment.

The HF and LF were hand-fed at four feeding levels at 25%, 50%, 75%, and 100% of apparent satiation to create a gradient in dietary energy intake. This feeding regime resulted in a 2 X 4 factorial design with eight treatment groups, namely HF25, HF50, HF75, HF100, and LF25, LF50, LF75, and LF100. Throughout the trial, the fish were fed three times daily at 9:30, 12:00, and 14:30 h. Each feeding session was completed within an hour. Apparent satiation at 100% for HF100 and LF100 was determined when the fish began to lose interest in feeding and when uneaten pellets were observed at the bottom of the tank. The other feeding levels of 25%, 50%, and 75% of apparent satiation were determined by adjusting proportionally using the averaged past 5-day feed intake amount of either HF100 or LF100.

### 2.3. Fish Sample Collection and Processing for Whole-Body Composition (WBC)

At the beginning of the trial, 50 fish from the initial population were euthanised by an overdose of Aqui-S (AQUI-S New Zealand Ltd., New Zealand) for the analysis of the initial carcass sample. At the end of the trial, all the fish from each tank were euthanised to determine the final BW and final carcass composition. Fish samples were kept at −20°C before processing. The sample processing for chemical analysis followed the methods reported by Bureau, Hua, and Cho [30] with modifications. Fish samples from each tank were thawed at four degrees overnight and autoclaved at 105°C for 40 min or until the samples were fully cooked. Cooked samples of each group were homogenised in a blender (HR3656/01, Philips) in small sub-batches and mixed using a KitchenAid (Benton Harbor, USA) stand mixer for 10 min. Mixed fish samples were freeze-dried and again homogenised before storing at −20°C until analysed.

### 2.4. Faecal Sample Collection and Processing for Digestibility

The fish in HF100 and LF100 were acclimated to their respective diets and dietary regimes over 7 days before the daily collection of faecal samples commenced, according to Cho, Slinger, and Bayley [31], with some modifications. Each experimental tank was connected to an individual faecal settling column. Both the tanks and settling columns were cleaned daily after 1 h from the last feeding to prevent contamination (e.g., uneaten feed) in the faecal samples. Overnight faecal samples settled in the columns were collected the next day at 9:00, before the first feeding, using a modified 350 mL syringe with an enlarged opening that fits 16 mm PVC pipe. The collected faecal samples were later transferred into a 250 mL conical centrifuge bottle and centrifuged using a refrigerated centrifuge (Centrifuge 5920 R, Eppendorf) at 3000 *g* for 15 min at 4°C. Daily wet faecal pellets were pooled by tank and stored at −20°C before freeze-drying. The freeze-dried faecal samples are sieved through a 0.3 mm screen mesh to remove any contaminating fish scales and stored at −20°C before chemical analysis.

### 2.5. Chemical Analyses

Diets, freeze-dried fish, and freeze-dried faecal samples were analysed for DM, ash, yttrium, CP, fat and gross energy (GE) content. The proximate composition of diets, fish, and faeces was analysed according to standard methods of analysis of the Association of Official Analytical Chemists [32] for the determination of DM (AOAC 930.15) and ash (AOAC 942.05). CP, fat, fibre, and yttrium content were determined by Pacific Lab Services according to AOAC 984.13 for the determination of CP (Kjeldahl; %N x 6.25), AOAC 920.39 for fat (Soxhlet), AOAC 978.10 for crude fibre (Fritted glass crucible method), and Pacific Lab Method 4.3, ICP-OES for yttrium. The gross GE content of the samples was measured using an automated bomb calorimeter (C 5000 Calorimeter, IKA) under the isoperibol setting. The measured GE of WBC had a good correlation with the calculated GE of WBC using the mean GE values of protein, fat, and carbohydrates (Figure S1, *R*^2^ = 0.85). However, the energy balance calculation for the mean values of sample GE, DE intake, ME intake, and RE were calculated for the mean GE values of protein, fat, and carbohydrates (23.6, 39.5, and 17.2 kJ g^−1^, respectively) according to Blaxter [33] for data consistency.

### 2.6. Growth and Nutrient Balance calculations

The growth rate was calculated using both the thermal growth coefficient (TGC) and the specific growth rate (SGR, % day^−1^). TGC is calculated according to the following equation [34, 35], and SGR is calculated according to the following equation [36]:

  $$\begin{array}{c} \text{TGC}=\left({\text{FBW}}^{\left(1/3\right)}-{\text{IBW}}^{\left(1/3\right)}\right)/\left(T\times d\right)\times 1000, \end{array}$$

  $$\begin{array}{c} \text{SGR}=\left(\text{ln FBW}-\text{ln IBW}\right)/d\times 100, \end{array}$$

where FBW and IBW are the final and initial BWs (g), *T* is the average temperature of the trial (°C), and *d* is the number of days.

The feed conversion efficiency (FCE, %) is calculated by the following:

  $$\begin{array}{c} \text{FCE}=\text{BWG}/\text{TFI }\times 100, \end{array}$$

where BWG (g) is the BW gain and TFI (g) is the total feed intake.

The apparent digestibility coefficients (ADC) for nutrients and energy in the diets were calculated according to the following equation [31]:

  $$\begin{array}{c} \text{ADC}=1-\left(\left(N_{F}/N_{D}\right)|\times |\left(Y_{D}/Y_{F}\right)\right), \end{array}$$

where *N*_D_ and *N*_F_ are the % nutrient (or kJ g^−1^ GE) of the diet and faecal matter, respectively. *Y*_D_ and *Y*_F_ are the % of Yttrium (digestion inert marker) of the diet and faecal matter, respectively.

Non-faecal losses of branchial and urinary nitrogen (BUN) losses were calculated as the difference between digestible nitrogen intake and nitrogen retention. The branchial and urinary energy (BUE) was estimated by multiplying BUN by 24.85, which is the energy content of 1 g of excreted nitrogen (in kJ), assuming that NH_3_-N is the only form of N excreted [37]:

  $$\begin{array}{c} \text{BUE}=\left(\text{DNI}-\text{RN }\right)\times 24.85, \end{array}$$

where DNI is digestible nitrogen intake (g) and RN is retained nitrogen (g).

The DE and ME were calculated according to NRC [28], where ME intake was calculated as the difference between DE intake and BUE.

The geometric mean BW (*W*_G_; in g) is calculated as $\sqrt{\text{IBW }\times \text{FBW}}$, from which the mean metabolic BW (MBW_G_; in kg^0.8^) is calculated as (*W*_G_/1000)^0.8^ with the assumption that the metabolic exponent of 0.8 is representative of the fish at this size [38].

The *k*_gDE_ was calculated from the slope of the regression of RE on DE intake. Maintenance energy requirement (DE_m_) was estimated by extrapolating the regression line to zero energy retention (where RE = 0).

### 2.7. Statistical Analysis

Data were analysed by using IBM SPSS Statistics version 27. A two-way ANOVA was used to investigate the effect of diet, feeding level, and the combined effects of diet and feeding levels on growth performance, apparent digestibility, nitrogen balance, and energy balance data. Values with *p*  < 0.05 were considered significant. For parameters with a significant interaction effect between diet and feeding level, Tukey's honestly significant difference (HSD) was used for the post hoc multiple comparison test. The linearity test was analysed using the R lm function from the stats package [29]. Linear regression between RE (in kJ kg^−0.8^ day^−1^) and DE intake (in kJ kg^−0.8^ day^−1^) was used to quantify the *k*_gDE_ of each diet using the model following Phan et al. [10]:

(1) $$\begin{array}{c} {\text{RE}}_{i}=\mu +\beta \times \text{DE}+e_{i}, \end{array}$$

where *μ* is the intercept, *β* is the energy utilisation efficiency, *e*_*i*_ is the error term, and *i* = n, with *n* = 16 for each diet.

The difference in the slopes of the regressions among different diets was tested for significance using a general linear model with RE as the dependent parameter, DE as a covariate, and diet as a fixed factor. If the interaction effect between diet and DE was significant (*p*  < 0.05), the slopes differed between diets.

Multiple regression of RE (in kJ kg^−0.8^ day^−1^) as a function of DE partition of DP, DF, and DC (in kJ kg^−0.8^ day^−1^) was used to estimate the DE utilisation efficiency of each macronutrient, adapted from Phan et al. [10] with minor modifications. Rather than using digestible macronutrients (in g kg ^−0.8^ day^−1^) in the multiple regression, the digestible macronutrients were multiplied by the mean GE values of protein, fat, and carbohydrates (23.6, 39.5, and 17.2 kJ g^−1^, respectively) according to Blaxter [33] to obtain the DE partition of DP, DF, and DC, before the multiple regression was analysed using the “Stepwise” method.

(2) $$\begin{array}{c} \text{NE}={\text{RE}}_{i}-\mu =\beta_{1}\times {\text{DE}}_{\text{DPi}}+\beta_{2}\times {\text{DE}}_{\text{DFi}}+\beta_{3}\times {\text{DE}}_{\text{DCi}}+e_{i}, \end{array}$$

where *μ* is the intercept, being an estimate for fasting heat production; *β*_1_, *β*_2_, *β*_3_ are the energy utilisation efficiency of partial DE as DP (*k*_NE;DP_), partial DE as DF (*k*_NE:DF_), and partial DE as DC (*k*_NE;DC_); *e*_i_ is the error term, and *i* = n, with *n* = 32.

## 3. Results

### 3.1. Effect of Diets and Feeding Levels on Growth Performance

The BW gain of juvenile Malabar snapper ranged from −0.5 g to 54.4 g among the treatments after 56 days (Table 2). By the end of the trial, the lowest feeding level group at 25% apparent satiation (HF25 and LF25) decreased in BW through the experimental duration. This indicated that the dietary energy intake for HF25 and LF25 was below the maintenance requirement for BW maintenance. On the other hand, HF100 and LF100 increased their BW by 92% and 77%, respectively. Among the growth performance parameters, the final BW was significantly affected by feeding level, diet, and their interaction (*p*  < 0.05; Table 2). Comparing the impact of diets against feeding levels, the difference in diet formulations had a minor influence on growth (*p*  < 0.05), whereas the change in feeding levels had a larger impact on BW gain, TGC, and FCE (*p* < 0.001; Table 2).

### 3.2. Effect of Diets and Feeding Level on Whole-Body Nutrient Composition

**Figure 1:**
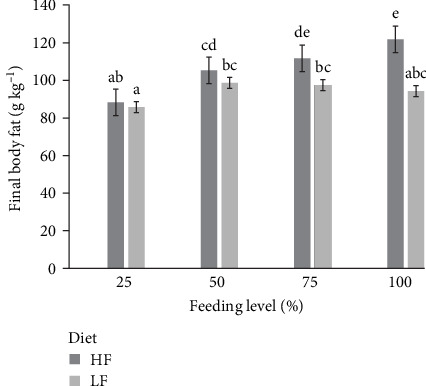
Mean ± SEM final body fat content (g kg^−1^, as is) of Malabar red snapper (*L. malabaricus*) fed with diet HF and LF at four different feeding levels (25%, 50%, 75%, and 100% of apparent satiation) for 56 days (*n* = 4). Mean values lacking a common letter superscript differ significantly (*p* < 0.05). HF, high fat; LF, low fat.

At the start of the experiment, the initial body fat content of the juvenile Malabar snapper was 116 g kg^−1^ (as is basis, Table S1). At reduced feeding levels of 25% and 50% apparent satiation, diet HF or LF did not cause any significant changes to final body fat content. However, at higher feeding levels of 75% and 100% apparent satiation, significantly higher final body fat content was observed in fish-fed diet HF than diet LF (*p* < 0.001; Figure 1). The final fat content of LF remained similar between the feeding levels (*p* > 0.05), while the final fat content of HF increased as feeding levels increased (*p* < 0.05), indicating an increase in fat deposition (FD) in the body.

### 3.3. Effect of Diets and Feeding Levels on Protein and Fat Energy Utilisation

The ADC of the treatment groups on 25%, 50%, and 75% feeding levels were estimated using the ADC of HF100 and LF100, respectively (Table S2). In other words, the analyses of digestible nutrients for groups fed below 100% apparent satiation were calculated based on the mean ADC values of either HF or LF. Most of the nutrient ADC between the two diets were similar (*p* > 0.05) except for fat, for which HF had higher apparent digestibility than LF (98.4% vs. 96.6%, *p* < 0.05). Ranking the average ADC of both HF and LF, fat has the highest digestibility at 97.5%, followed by protein, GE, nitrogen-free extract (NFE), DM, carbohydrates, and lastly ash at 18.25%.

The outcomes were intended, as the experimental design, to create large differences in RE among the eight treatment groups. Protein gain and protein deposition were altered by feeding level, whereas fat gain and fat deposition are affected by diet, feeding level, and their interactions (*p* < 0.05, Table 3). The average values of RE as protein and RE as Fat were 25.9 kJ kg^−0.8^ day^−1^ and 26.1 kJ kg^−0.8^ day^−1^ at 100% apparent satiation, respectively (Table 3).

**Figure 2:**
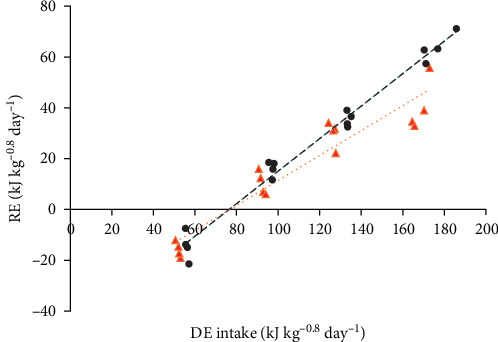
Relationship between retained energy (RE) and digestible energy intake (DE) for Malabar red snapper (*L. malabaricus*) fed with either diets HF or LF (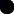 HF: RE = −48.95 [SE 2.89] + 0.64 [SE 0.023] DE, *R*^2^ = 0.98; 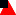 LF: RE = −37.74 [SE 4.96] + 0.49 [SE 0.042] DE, *R*^2^ = 0.91) for 56 days. By extrapolating to zero energy retention (RE = 0), the estimated digestible energy requirements for maintenance were 76.5 kJ kg^−0.8^ day^−1^ and 76.9 kJ kg^−0.8^ day^−1^ for HF and LF, respectively. HF, high fat; LF, low fat.

**Figure 3:**
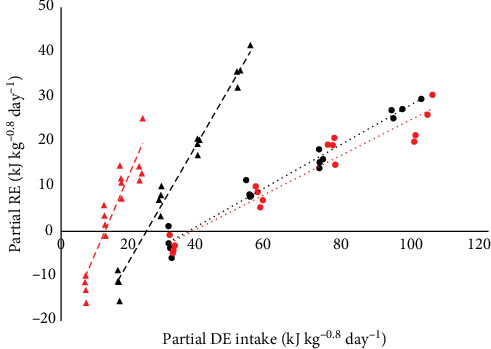
Relationship between retained energy (RE) partitioned as nutrients (either protein or fat) as a function of digestible energy (DE) intake partitioned as nutrients (either protein or fat) of *L. malabaricus* fed either diet HF (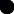 RE as Prot = −16.078 [SE 1.459] + 0.451 [SE 0.012] DE_DP_, *R*^2^ = 0.969; 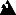 RE as fat = −32.904 [SE 1.753] + 1.373 [SE 0.049] DE_DF_, *R*^2^ = 0.982) or diet LF (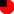 RE as Prot = −15.014 [SE 2.136] + 0.407 [SE 0.040] DE_DP_, *R*^2^ = 0.929; 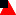 RE as fat = −22.710 [SE 3.172] + 1.848 [SE 0.202] DE_DF_, *R*^2^ = 0.856) for 56 days. HF, high fat; LF, low fat.

The first objective of this study was to evaluate the effect of two isonitrogenous diets with contrasting fat and carbohydrate content on the relationship between DE intake (in kJ kg^−0.8^ day^−1^) and RE (in kJ kg^−0.8^ day^−1^) in Malabar snapper. DE intake requirements for maintenance (DE_m_) were estimated (RE = 0) to be 76.5 kJ kg^−0.8^ day^−1^ and 76.9 kJ kg^−0.8^ day^−1^ for HF and LF, respectively (Figure 2). Based on the linear regression slope, *k*_gDE_ can be estimated. LF, which had higher dietary carbohydrates and lower fat, had significantly lower *k*_gDE_ compared to HF (49% vs. 64%; *p* < 0.001). Further assessment of the DE utilisation was examined through the relationships between partitioning of RE (as either protein or lipid) as a function of partial DE (intake as either protein or lipid) by diets, as visualised in Figure 3. The following regression equations of the partial DE utilisation efficiency of protein (*k*_P_) or fat (*k*_F_) were obtained for both HF and LF independently.

(3) $$\begin{array}{c} \text{HF};\text{RE as Prot}=-16.078\;\left(\text{SE }1.459\right)+0.451\;\left(\text{SE }0.012\right)\;{\text{DE}}_{\text{DP}},R^{2}=0.969, \end{array}$$

(4) $$\begin{array}{c} \text{HF};\text{RE as Fat }=-32.904\;\left(\text{SE }1.753\right)+1.373\;\left(\text{SE }0.049\right)\;{\text{DE}}_{\text{DF}},R^{2}=0.982, \end{array}$$

(5) $$\begin{array}{c} \text{LF};\text{RE as Prot}=-15.014\;\left(\text{SE }2.136\right)+0.407\;\left(\text{SE }0.040\right)\;{\text{DE}}_{\text{DP}},R^{2}=0.929, \end{array}$$

(6) $$\begin{array}{c} \text{LF};\text{RE as Fat}=-22.710\;\left(\text{SE }3.172\right)+1.848\;\left(\text{SE }0.202\right)\;{\text{DE}}_{\text{DF}},R^{2}=0.856. \end{array}$$

The reciprocal value of the regression coefficient in each regression (either 1/*k*_P_ or 1/*k*_F_) estimates the DE cost of protein deposition or fat deposition in juvenile Malabar snapper above maintenance. The average costs of protein deposition and fat deposition are 2.33 kJ per kJ and 0.62 kJ per kJ, respectively. It is important to note that the calculation for DE cost for fat deposition only accounted for DF energy, while factually, fat could also be deposited from excess DP and DC. The regression equations were compared using a general linear model with RE as the dependent variable, DE as the covariate, and diet as a fixed factor. The *k*_P_ between diets Equations (3) and (5) were found to be similar (*p* = 0.68). On the other hand, the *k*_F_ between diets Equations (4) and (6) were significantly different (*p* < 0.001).

The next objective of this study was to measure the energy utilisation efficiency of digestible macronutrients (i.e., DP, DF, and DC) using the multifactorial approach based on NE. By conducting multiple linear regression (method = Stepwise) between RE (in kJ kg^−0.8^ day^−1^) and the partial DE intake of DP, DF, and DC (in kJ kg^−0.8^ day^−1^), the following RE equation was estimated.

(7) $$\begin{array}{c} \text{RE}=-43.271\;\left(\text{SE }3.016\right)+0.737\;\left(\text{SE }0.056\right)\;{\text{DP}}_{\text{DE}}+0.683\;\left(\text{SE }0.103\right)\;{\text{DF}}_{\text{DE}},R^{2}=0.95. \end{array}$$

**Figure 4:**
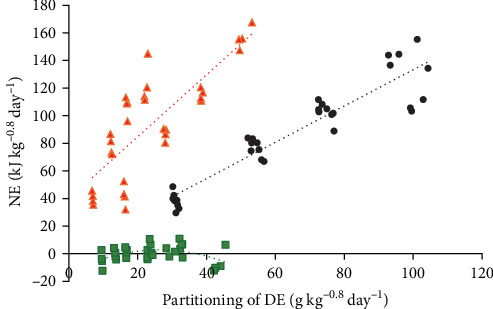
Relationship between net energy (NE) and digestible energy (DE) partitioned as digestible protein (DE_DP_), digestible fat (DE_DF_), and digestible carbohydrate (DE_DC_) in *L. malabaricus*. The NE values corresponding to DE_DP_ are corrected for variation in DE_DF_ and DE_DC_, the NE values corresponding to DE_DF_ are corrected for DE_DP_ and DE_DC_, and the NE values corresponding to DE_DC_ are corrected for variation in DE_DP_ and DE_DF_. The calculations were conducted using Equation (7) is as follows: the measured retained energy for each data point in the data set was added with the estimated fasting heat production (intercept) to obtain the NE value. The NE values are then corrected towards zero DE_DF_ and DE_DC_ (where DE_DC_ = 0) to visualise the effect of DE_DP_ on NE, 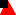; zero DE_DP_ and DE_DC_ (where DE_DC_ = 0) to visualise the effect of DE_DF_ on NE, 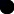; and zero DE_DP_ and DE_DF_ to visualise the effect of DE_DC_ on NE, 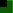.

The energy utilisation efficiencies of DP (*k*_NE;DP_) and DF (*k*_NE;DF_) were 73.7% and 68.3%, respectively. From the multiple regression Equation (7), both changes in DP and fat were significantly associated with the changes in RE, without DCs. The relationship between NE and DE of micronutrients derived from the estimated Equation (7) can be visualised in Figure 4. A linearity test on both DP and DF on NE observed no significant polynomial effect (*p* > 0.05); however, a significant quadratic component or curvilinear relationship was found for DC (*p* < 0.01). Because DC does not contribute significantly to RE (*p* < 0.001) in this study, forcing the DC linear or quadratic component on the regression model has resulted in a nonsensical equation between RE and the digestible macronutrients.

## 4. Discussion

### 4.1. Energy Utilisation Efficiency of Malabar Snapper Based on Factorial Approach

Fish require an exogenous source of energy above their maintenance requirements for growth. This dietary energy in the form of protein, fat, or carbohydrates can be acquired through feeds, but the intake of nutrients must be digestible and utilised for various metabolic pathways that result in protein and fat deposition (growth). Nutrient and energy requirement values are needed to formulate aquafeed to meet energy demands for growth. Studies on the energy balance between dietary energy intake, expenditure (cost), and retention (gain) are useful for understanding dietary nutrient utilisation and developing bioenergetic approaches or models to estimate energy demands [39].

Fish energy demands for maintenance and growth are commonly assessed using the factorial approach based on DE intake. The DE requirement for maintenance (DE_m_) across 14 fish species has been reviewed by Schrama et al. [8], who reported a range of DE_m_ values between 27 and 88 kJ kg^−0.8^ day^−1^ for fish. In the present study, the average DE_m_ of Malabar snapper was found to be 76.7 kJ kg^−0.8^ day^−1^ under the experimental conditions, which was within the DE_m_ expected range for fish. However, the DE_m_ of Malabar snapper was much higher compared to other tropical species, such as snakehead, which ranged from 7 to 48 kJ kg^−0.8^ day^−1^ [10], barramundi, which ranged from 8.7 to 29.8 kJ kg^−0.8^ day^−1^ [9] and Nile tilapia, which ranged from 62 to 67 kJ kg^−0.8^ day^−1^ [8]. A fish with a high maintenance energy requirement implies that more dietary energy is needed to support basal metabolism before the energy can be made available for growth [40].

The *k*_gDE_ was evaluated based on the regression of RE on DE intake. The current estimates of *k*_gDE_ in Malabar snapper ranging from 49% to 64%, are comparable to the estimated *k*_gDE_ of other fish species such as barramundi, which range from 51% to 79% [6, 9, 14], common carp ranging between 59% and 66% [14], European seabass which ranges from 64% to 67% [41], snakehead which ranges from 45% to 56% [10] and Nile tilapia which ranges from 56% to 66% [13]. In comparison to rainbow trout and catfish, Malabar snapper had lower *k*_gDE_ than rainbow trout [13, 42] and African catfish [11]. The higher *k*_gDE_ for rainbow trout and African catfish indicated that they were better at utilising dietary nutrients for growth compared to Malabar snapper. The current study was consistent with the above studies that determined the factorial approach based on DE could be biased and could be influenced by diet macronutrient composition. Through the factorial analyses of HF and LF, the *k*_gDE_ of Malabar snapper was affected by dietary nutrient composition, where the lower energy diet with higher carbohydrates, lower fats, and lower fish meal content resulted in poorer *k*_gDE_. The low *k*_gDE_ associated with diets containing high carbohydrate content could be linked to the nature of carnivorous fish, which have a limited ability to utilise carbohydrates as an energy source for metabolic activity and growth [43].

### 4.2. Protein and Fat Utilisation Efficiencies of Malabar Snapper Based on Factorial Approach

The *k*_P_ or *k*_F_ refers to the amount of dietary DP or fat (g kg^−0.8^ day^−1^) needed to gain a gram of protein or fat in fish. Alternatively, the utilisation efficiencies of DP or fat can also be expressed in energy function (kJ kg^−0.8^ day^−1^) based on the energy equivalent values of protein and fats. In this study, the *k*_p_ values of Malabar snapper were estimated at 0.451 and 0.407 for HF and LF, respectively, and no significant difference was observed between the two diets (Figure 3). In comparison with other carnivore fish, the protein utilisation efficiency of Malabar snapper was at the lower end of the spectrum for barramundi, which had *k*_p_ values that ranged from 0.42 to 0.58 [6, 9] and was poorer compared to the reported *k*_p_ values of 0.526, 0.532, and 0.559 for European seabass, gilthead seabream and white grouper (*Epinephelus aeneus*), respectively [3]. The energy cost of protein deposition can be calculated from the reciprocal value of *k*_P_. The estimated 1/*k*_P_ of Malabar red snapper was determined to be 2.22 and 2.46 kJ per kJ of protein deposition above maintenance for HF and LF, respectively. These were similar to the protein energy cost of barramundi-fed diets with a high dietary carbohydrate inclusion level of 33.6 g 100 g^−1^, having a 1/*k*_P_ value of 2·43 [9]. Given that the carbohydrate content of LF used in this study (33.0 g 100 g^−1^) was similar to the high starch-containing diet used by Glencross et al. [9], our study agreed with the findings of other studies that dietary carbohydrates were likely utilised less efficiently than protein as an energy source for growth in carnivorous fish.

In the present study, the *k*_F_ for HF and LF were estimated as 1.373 and 1.848, respectively. Utilisation efficiencies greater than 1 indicate higher fat deposition than dietary fat intake, and we infer that lipids were being synthesised from other macronutrients and stored as fat in the fish. These high *k*_F_ supported observations made in this study that Malabar is a fish with high adiposity (Figure 1). The significantly higher *k*_F_ observed for LF with LF content indicated the availability of excess energy (other than dietary fats), likely from the higher DCs, which were synthesised and deposited as fats. The 1/*k*_F_ in this study was estimated between 0.54 and 0.72 kJ per kJ of fat energy deposited. This was similar to the range of values reported for rainbow trout and barramundi, from 0.65 to 0.93 [9, 44]. In comparison to other fish species, considerably less dietary energy was needed to store fat in Malabar snapper compared to gilthead seabream, European seabass, white grouper, and carp, which had energy costs of lipid deposition above one and ranging from 1.10 to 1.39 [3, 45]. The energy cost of lipid deposition below one implied that the fat energy accumulation from dietary fat intake was a highly efficient process in Malabar snapper, similar to other carnivorous species [8, 9]. The significantly lower 1/*k*_F_ observed for LF with higher carbohydrate content and lower fat content promoted lipogenesis activity in Malabar snapper similar to barramundi [46] and European seabass [47], where dietary carbohydrates were digested, converted, and stored as fat.

### 4.3. Protein and Fat Utilisation Efficiencies of Malabar Snapper Based on Multifactorial Approach

**Figure 5:**
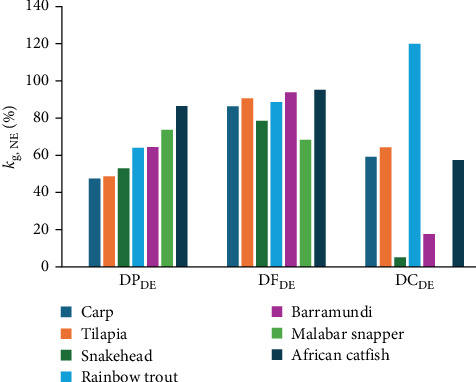
Net energy utilisation efficiencies (*k*_g,NE_) of energy partition of digestible protein (DP_DE_), Fat (DF_DE_), and carbohydrate (DC_DE_) in carp, tilapia, snakehead, rainbow trout, barramundi, African catfish, and Malabar snapper using linear relationship between retained energy and digestible energy intake of protein, fat, and carbohydrate. The coefficient data of barramundi and carp were obtained from Phan et al. [14], trout and tilapia were from Schrama et al. [13], snakehead was from Phan et al. [10], African catfish was from Phan et al. [11], and Malabar Snapper from this study.

The conventional factorial approach based on DE intake typically assumes that the estimated *k*_gDE_ is independent of the ratio of macronutrient composition in the diet. As an alternative to the factorial approach, Schrama et al. [13] have adopted the NE evaluation system from pig nutrition [12] and developed NE equations related to RE to the digestible macronutrient for fish. The NE equation and multifactorial approach have since been applied to tilapia and trout [13], barramundi and common carp [14], snakehead [10], and African catfish [11]. Evaluation of the partial energy utilisation efficiency (*k*_g, NE_) values of digestible macronutrients usually required the coefficient values derived from the relationship between NE and digestible macronutrients to be divided by the GE values of those macronutrients (23.6, 39.5, and 17.2 kJ g^−1^ for CP, fat, and carbohydrates, respectively). In this study, the original equation was modified slightly by studying RE to the DE of macronutrients to reflect the partial energy utilisation efficiencies coefficient values within the equation without needing additional conversion. Instead of investigating the relationship between NE and digestible macronutrients (g kg^−0.8^ day^−1^), the energetic values of DP, DF, and DC (kJ kg^−0.8^ day^−1^) were used. Estimates of energy utilisation efficiencies of DP (*k*_NE:DP_), DF (*k*_NE:DF_), and DC (*k*_NE:DC_) between the fish species were compared in Figure 5 [48]. The estimated *k*_NE:DP_ based on the linear relationship for Malabar snapper was 73.6%. This value was the second highest among the species investigated but lower than African catfish, which had a utilisation efficiency of 86% [11]. From the comparison, Malabar snapper seemed very efficient at utilising DP energy for growth compared to other fish species (Figure 5); however, the protein retention efficiency values in this study range from minus 10% to 28% (Table 3). These values are much lower compared to barramundi values, which range from 37% to 55% [9], snakehead 43%–54% [10], and African catfish 61%–73% [11]. The impact of low protein gain efficiency on growth performance is evident from the slow growth rates observed (Table 2). All things considered, this suggests that Malabar snapper can utilise DP effectively but has a strong preference to use amino acids for maintaining its metabolic requirements. In other words, given the relatively high DE_m_ (Figure 2), a large proportion of the DP energy has been used to meet the energetic needs for maintenance.

The estimated *k*_NE:DF_ of Malabar snapper was 68.3%. This value was the lowest among other fish species investigated (Figure 5). However, this relatively lower *k*_NE:DF_ value does not restrict fat retention efficiency, which ranged from minus 181% to 72%. Under restricted dietary intake conditions below maintenance requirements (HF25 and LF25), stored fat was prioritised over body protein to be metabolised as fuels for energy (Table 3), demonstrating the protein-sparing effect of lipids. Above maintenance energy requirements (HF100 and LF100), fat retention efficiency was greater than protein retention efficiency, which further suggested that dietary fats were deposited while dietary proteins were preferentially metabolised to provide energy for maintenance.

The apparent digestibility of carbohydrates in the present study was up to 83% (Table S2). However, the *k*_NE:DC_ value could not be estimated through the multi-regression analyses in this study. The *k*_NE:DC_ values derived linearly from other fish species were estimated to range from 5% for snakehead to 120% for rainbow trout (Figure 5). This inability to estimate the *k*_NE:DC_ value indicated that Malabar snapper is unable or has a very limited capacity to metabolise carbohydrates efficiently for growth. Alternatively, this also suggested that the experimental design of two dietary carbohydrate levels and four feeding levels was insufficient to elucidate the energy utilisation of digestible carbohydrates, and further studies with more inclusion levels of digestible carbohydrates may be needed.

### 4.4. Adiposity of Malabar Snapper

In this study, Malabar snapper fed with higher dietary fat content at apparent satiation had increased in fat digestibility (Table S1), a higher body fat composition of 122 g kg^−1^ (Figure 1 and Table S1), improved BW gain and growth rates (Table 2). This positive effect of high dietary fat intake on fat digestibility has previously been reported for African catfish [11]. However, there are discrepancies among fish species where the level of dietary fat intake does not affect fat digestion, for example, in Atlantic halibut (*Hypoglossus hippoglossus*) [49], Nile tilapia [8], and snakehead [10] but negatively impacts fat digestion in common carp [14, 50]. Direct assessment of the effect of dietary fat intake among fish species can be challenging due to differences in the dietary macronutrient contents, nutrient apparent digestibility, and utilisation efficiencies. The proportion of fat retention in the body can be represented by the fat gain to protein gain ratio. In the current study, the fat gain: protein gain was 0.79 g g^−1^ at the highest DF intake of 1.3 g kg^−0.8^ day^−1^ (Table 3). In comparison with other carnivorous species at the same DF intake level, the proportion of fat retention in barramundi and snakehead was 0.59 and 0.57 g g^−1^, respectively, and the ratio did not increase even at higher DF intakes of 2 or 3 g kg^−0.8^ day^−1^ [9, 10]. For other fish species, the average proportion of fat gain is higher for rainbow trout and common carp at 0.95 g g^−1^ and 1.1 g g^−1^, respectively [14, 30]. These data support the fact that the Malabar snapper is a carnivorous species and has a stronger preference to gain protein over fat. At the same dietary energy level, Malabar snapper could deposit greater amounts of fat than either barramundi or snakehead, indicating a lower energy demand for dietary fat. Further studies with a higher DF intake above 1.3 g kg^−0.8^ day^−1^ are needed to determine if Malabar snapper could have a higher proportion of fat ratio (above 0.79 g g^−1^). Moreover, it would be interesting to evaluate whether the body fats are deposited in the muscle fillets, like salmonid species [51, 52], or accumulated in the visceral adipose tissues.

### 4.5. Growth Performance of Malabar Snapper

In this study, the juvenile Malabar snapper growth rate between 57 and 111 g was slower than expected, even though both HF and LF were designed to meet or exceed the known nutritional requirements of other snapper species [21–26]. The average snapper SGR and TGC fed 100% apparent satiation were 1.09% day^−1^ and 0.53, respectively (Table 2). Direct comparisons between growth rates across or within species are challenging since smaller fish always have higher relative growth rates than larger fish. The growth rates of juvenile Malabar snapper in this study are comparable to the growth performance of some snapper species; for example, an average SGR of 0.93% day^−1^ and TGC of 0.46 have been reported for juvenile spotted rose snapper (*L. guttatus*) cultured in floating net cages for a year [53] and an average SGR of 1% day^−1^ under experimental conditions [54]. But in comparison to juvenile mangrove red snapper (*L. argentimaculatus*) with an initial BW of 12.3 g, SGR up to 2.3% day^−1^ was observed [25]. Very few growth studies on Malabar snapper are available, and the only nutritional study found was on fingerlings (<16 g), which reported an SGR of 3.56% day^−1^ when fed a commercial diet and reared at 30°C [55]. In comparison to other species, SGR above 2.6% day^−1^ has been reported for juvenile barramundi [56] and Nile tilapia [57]. Possible reasons for the slower growth rates compared to other juvenile fish could be related to feed quality issues (i.e., the presence of anti-nutritional factors or ingredient palatability) or environmental factors such as different farming conditions. The slower-than-expected growth rate might also result from experimental diets formulated based on other snappers' requirements and warrants further studies to determine Malabar snapper nutrient requirements.

## 5. Conclusion

The current study further supports the findings of previous studies that the energy utilisation efficiency of DE intake for growth is affected by dietary macronutrient composition. Like most carnivorous fish, Malabar snapper utilises DP and fat efficiently, but not DCs. Fish fed with LF had lower energy utilisation efficiency for growth and higher partial energy utilisation efficiency of DF. This implies an increased rate of lipogenesis activity, in which high dietary carbohydrates (at low levels of dietary fat) are likely not utilised for growth but are converted and accumulated as fats in the body. Although further studies are needed to determine the acceptable level of dietary carbohydrate content, one should be wary of the negative effects of dietary carbohydrate supplementation when formulating diets for this species. The maintenance energy requirement of Malabar snapper was estimated to be 76.7 kJ kg^−0.8^ day^−1^, and the utilisation efficiencies of DP and DF were estimated to be 73.6% and 68.3%, respectively. At DE intake levels below maintenance, Malabar snapper has a strong preference to utilise DF over protein for metabolism, demonstrating a protein-sparing effect from lipids. In contrast, given the higher efficiency of fat retention than protein retention, protein is likely used before fat when energy intake is above maintenance. This high maintenance energy requirement and the strong preference to utilise dietary protein for growth reinforce the fact that Malabar snapper is a carnivore fish that requires high levels of DP in its diet and suggest future studies to develop the protein and energy requirements of this species. The relatively slow growth rates and low protein retention efficiency observed highlighted the need to develop an optimised diet for this aquaculture species to improve its productivity.

## Figures and Tables

**Table 1:** Diet formulation and analysed nutrient composition of HF and LF fed to Malabar red snapper (*L. malabaricus*).

Composition	Diet	
	HF	LF
Diet formulation (g kg^−1^, as is)		
Fish meal^a^	450	300
Wheat flour^b^	136	181
Wheat gluten^c^	0	60
Soybean meal^d^	150	200
Soybean concentrate^e^	50	80
Corn gluten Meal^f^	80	100
Corn starch^g^	20	20
Sardine oil^h^	85	30
Vitamin mix^†^	7.5	7.5
Mineral mix^‡^	7.5	7.5
MDCP	10	10
Choline chloride, 60% corn cob^i^	1	1
Vitamin C, Stay C-35^j^	1	1
Moldinhibitor^k^	0.8	0.8
Antioxidant^l^	0.2	0.2
Yttrium oxide^m^	1	1
Nutrient composition (g 100 g^−1^, DM)		
DM (as is)	93.3	92.5
Crude protein (CP)	50.3	51.1
Crude fat	15.2	6.5
Fibre	0.9	1.5
Carbohydrate^§^	23.6	33.0
Crude ash	10.0	8.6
Gross energy^¶^ (kJ g^−1^)	21.9	20.3
Digestible protein (DP)	47.3	47.8
Digestible energy (DE kJ g^−1^)	20.4	18.7
DP:DE (g MJ^−1^)	23.2	25.6

Abbreviations: DM, dry matter; HF, diet high-fat; LF, diet low-fat.

^a^FF Classic, FF SKAGEN A/S, Denmark.

^b^Little Shephard Brand, Prima Ltd., Singapore.

^c^Vital wheat gluten, Malindra group, USA.

^d^Hipro soybean meal, Argentina.

^e^X-soy 200, CJ selecta, Brazil.

^f^Cargill, USA.

^g^Daesang corporation, Korea.

^h^Sardine fish oil, Indonesia.

^i^Choline Chloride, 60% corn cob, Shandong Jujia Biotech Co., Ltd., China

^j^L-ascorbate-2-polyphosphate, Rovimix Stay-C 35, DSM, Netherlands.

^k^Funginat, Norel animal nutrition, Singapore

^l^Butylated hydroxytoluene (BHT), 2%; Butylated hydroxyanisol (BHA), 0.5%; Ethoxyquin, 3.2%; Haltox, Zargo, Singapore.

^m^Yttrium (III) oxide, 99.9%, CAS: 1314-36-9, Thermo Fisher Scientific, Singapore.

^†^Provide per kg of diet: vitamin A, 2000 MIU; vitamin D3, 400 MIU; vitamin E, 20 g; vitamin B2, 5 g; vitamin K3, 2 g; nicotinic acid, 15 g; calcium pantothenate, 10 g; folic acid, 0.5 g; vitamin B1, 2 g; vitamin B6, 2 g; vitamin B12, 10 mg.

^‡^Provide per kg of diet: iron, 100 g; copper 10 g; manganese, 70 g; zinc, 80 g; cobalt, 300 mg; iodine, 1000 mg.

^§^Calculated as: 100% −sum (CP% + Fat% + Ash% + Moisture%).

^¶^Calculated from mean GE values of protein, fat, and carbohydrate (23.6, 39.5, and 17.2 kJ g^−1^, respectively).

**Table 2:** Growth performance data of Malabar red snapper (*L. malabaricus*)^†^ fed two diets (HF, LF) at four feeding levels (FL) after 56 days trial period (*n* = 4).

Parameters	Diet	Feeding level (FL)				Pooled SEM	*p*-Value		
		25%	50%	75%	100%		Diet	FL	Diet X FL
Final weight (g)	HF	57.2^a^	75^b^	90.3^c^	111.2^d^	5.24	*⁣* ^*∗*^	*⁣* ^*∗∗∗*^	*⁣* ^*∗*^
	LF	57.5^a^	72.1^b^	88.8^c^	102.3^e^				

Weight gain (g)	HF	−0.6	17.0	32.5	53.4	5.29	*⁣* ^*∗*^	*⁣* ^*∗∗∗*^	N.S
	LF	−0.5	14.3	30.9	44.5				

TGC	HF	0.0	0.2	0.4	0.6	0.1	*⁣* ^*∗*^	*⁣* ^*∗∗∗*^	N.S
	LF	0.0	0.2	0.4	0.5				

SGR (% day^−1^)	HF	0.0	0.5	0.8	1.2	0.12	*⁣* ^*∗*^	*⁣* ^*∗∗∗*^	N.S
	LF	0.0	0.4	0.8	1.0				

TFI (g)	HF	16.7	32.3	47.9	68.4	2.33	N.S	*⁣* ^*∗∗∗*^	N.S
	LF	17.1	33.1	49.3	69.5				

FCE (%)	HF	0.0	0.5	0.7	0.8	0.16	N.S	*⁣* ^*∗∗∗*^	N.S
	LF	0.0	0.4	0.6	0.6				

Survival (%)	HF	85	80	89	94	0.14	N.S	*⁣* ^*∗*^	N.S
	LF	82	83	88	93				

*Note:* Values are mean (*n* = 4). *⁣*^*∗∗∗*^*p* < 0.001; *⁣*^*∗*^*p* < 0.05; N.S, non-significantly different (two-way ANOVA).

Abbreviations: FCE, feed conversion efficiency; SGR, specific growth rate; TFI, total feed intake; TGC, thermal growth coefficient.

^a,b,c,d,e^For parameters with significant interaction effect between diet and feeding level. Mean values lacking a common superscript differ significantly (*p* < 0.05) (Tukey's HSD).

^†^Initial body weight (g) = 57.84 (SD 0.13).

**Table 3:** Nutrient intake and energy balance of Malabar snapper (*L. malabaricus*), fed two different diets (HF, LF) at four different feeding levels (FL) for 56 days (*n* = 4).

Parameters	Diet	Feeding levels (FL)				Pooled SEM	*p*-Value		
		25%	50%	75%	100%		Diet	FL	Diet X FL
DP (g kg^−0.8^ day^−1^)	HF	1.3	2.2	3.1	4.1	0.12	*⁣* ^*∗∗∗*^	*⁣* ^*∗∗∗*^	N.S
	LF	1.3	2.4	3.2	4.3				

DF (g kg^−0.8^ day^−1^)	HF	0.4^c^	0.7^e^	1.0^f^	1.3^g^	0.03	*⁣* ^*∗∗∗*^	*⁣* ^*∗∗∗*^	*⁣* ^*∗∗∗*^
	LF	0.2^a^	0.3^b^	0.4^c^	0.6^d^				

DC (g kg^−0.8^ day^−1^)	HF	0.6^a^	1^c^	1.3^d^	1.8^e^	0.08	*⁣* ^*∗∗∗*^	*⁣* ^*∗∗∗*^	*⁣* ^*∗∗∗*^
	LF	0.8^b^	1.4^d^	1.9^f^	2.5^g^				

Prot_gain_ (g kg^−0.8^ day^−1^)	HF	−0.1	0.4	0.7	1.2	0.18	NS	*⁣* ^*∗∗∗*^	N.S
	LF	−0.1	0.3	0.8	1.0				

Fat_gain_ (g kg^−0.8^ day^−1^)	HF	−0.3^a^	0.2^b,c^	0.5^e^	0.9^f^	0.14	*⁣* ^*∗∗∗*^	*⁣* ^*∗∗∗*^	*⁣* ^*∗∗∗*^
	LF	−0.3^a^	0.1^b^	0.3^c,d^	0.4^d,e^				

GE intake (kJ kg^−0.8^ day^−1^)	HF	60	105	144	190	4.51	*⁣* ^*∗*^	*⁣* ^*∗∗∗*^	N.S
	LF	57	100	137	183				

DE intake (kJ kg^−0.8^ day^−1^)	HF	56	97	134	176	4.98	*⁣* ^*∗*^	*⁣* ^*∗∗∗*^	N.S
	LF	52	92	126	168				

BUE loss (kJ kg^−0.8^ day^−1^)	HF	5.6^a^	7.4^b^	9.6^c^	12^d^	0.71	*⁣* ^*∗∗∗*^	*⁣* ^*∗∗∗*^	*⁣* ^*∗*^
	LF	5.8^a^	8.1^b^	9.7^c^	13^e^				

ME intake (kJ kg^−0.8^ day^−1^)	HF	51	90	124	165	4.79	*⁣* ^*∗∗∗*^	*⁣* ^*∗∗∗*^	N.S
	LF	46	84	117	155				

RE (kJ kg^−0.8^ day^−1^)	HF	−14^a^	16^b,c^	35^c,d^	64^e^	9.15	*⁣* ^*∗∗∗*^	*⁣* ^*∗∗∗*^	*⁣* ^*∗*^
	LF	−16^a^	10^b^	30^c,d^	41^d^				

PD (RE as Prot) (kJ kg^−0.8^ day^−1^)	HF	−2.8	8.9	16	27	4.26	N.S	*⁣* ^*∗∗∗*^	N.S
	LF	−3.2	7.8	19	25				

FD (RE as fat) (kJ kg^−0.8^ day^−1^)	HF	−12^a^	7.2^b,c^	19^d^	36^e^	5.72	*⁣* ^*∗∗∗*^	*⁣* ^*∗∗∗*^	*⁣* ^*∗*^
	LF	−13^a^	2.5^b^	11^b,c^	16^c,d^				

FD:PD (J J^−1^)	HF	—	0.84	1.22	1.33	0.32	*⁣* ^*∗∗∗*^	*⁣* ^*∗*^	N.S
	LF	—	0.28	0.60	0.65				

Fat_gain_: Prot_gain_ (g g^−1^)	HF	—	0.50	0.73	0.79	0.19	*⁣* ^*∗∗∗*^	*⁣* ^*∗*^	N.S
	LF	—	0.17	0.36	0.39				

Prot_ret_ efficiency (%)	HF	−9	17	22	28	7.89	N.S	*⁣* ^*∗∗∗*^	N.S
	LF	−10	14	24	24				

Fat_ret_ efficiency^†^ (%)	HF	−72^b^	26^c,d^	50^c,d^	72^d^	33.2	*⁣* ^*∗*^	*⁣* ^*∗∗∗*^	*⁣* ^*∗∗∗*^
	LF	−181^a^	21^c^	67^c,d^	71^d^				

RE efficiency (%)	HF	−26	17	26	36	8.62	*⁣* ^*∗*^	*⁣* ^*∗∗∗*^	NS
	LF	−30	11	24	24				

*Note:* Values are mean (*n* = 4). *⁣*^*∗∗∗*^*p* < 0.001; *⁣*^*∗*^*p* < 0.05; N.S, non-significantly different (two-way ANOVA).

Abbreviations: BUE, branchial urinary energy; DC, digestible carbohydrate intake; DE, digestible energy; DF, digestible fat intake; DP, digestible crude protein intake; Fat_gain_, body fat gain; Fat_ret_ efficiency, fat retention efficiency, body fat gain X 100/digestible fat intake; FD, partial fat deposition from RE; GE, gross energy; ME, metabolisable energy; PD, partial protein deposition from RE; Prot_gain_, body protein gain; Prot_ret_ efficiency, protein retention efficiency, body protein gain X 100/digestible protein intake; RE, retained energy; RE efficiency, body retained energy X 100/digestible energy intake.

^a,b,c,d,e^For parameters with significant interaction effects between the combined effects of diet and feeding level. Mean values lacking a common superscript differ significantly (*p* < 0.05) (Tukey's HSD).

^†^Calculation for fat retention efficiency only accounts for digestible fat intake. It is important to note that body fat can also be derived from excess digestible protein and carbohydrates.

## Data Availability

Data will be made available from the corresponding author upon reasonable request.

## Supporting Information

Supporting Information Table S1. Initial and final body composition of juvenile Malabar red snapper fed two diets (HF and LF) at four feeding levels (FL) for 56 days (*n* = 4). Table S2. Mean apparent digestibility coefficient (ADC) of dietary nutrient intake in Malabar red Snapper (*L. malabaricus*) for diets HF and LF during the 56-day experimental period (*n* = 4). Figure S1. Relationship between measured GE (kJ g^−1^) and calculated GE (kJ g^−1^) values of whole-body composition (WBC).
