# Supplementary material for: Evaluation of Energy Utilisation Efficiencies of Digestible Macronutrients in Juvenile Malabar Snapper (Lutjanus malabaricus) Reveals High Protein Requirement for Optimal Growth Using Both Factorial and Multifactorial Approaches
Source: Aquac Nutr. 2024 Dec 21;2024:5467206. doi: 10.1155/anu/5467206 (PMC11681985; doi:10.1155/anu/5467206)
Supplement: Supporting Information — Table S1. Initial and final body composition of juvenile Malabar red snapper fed two diets (HF and LF) at four feeding levels (FL) for 56 days (n = 4). Table S2. Mean apparent digestibility coefficient (ADC) of dietary nutrient intake in Malabar red Snapper (L. malabaricus) for diets HF and LF during the 56-day experimental period (n = 4). Figure S1. Relationship between measured GE (kJ g−1) and calculated GE (kJ g−1) values of whole-body composition (WBC). [file 5467206.f1.pdf]

Supplementary Table S1. Initial and final body composition of juvenile Malabar red snapper fed two diets (HF and LF) at four feeding levels (FL) for 56 days (n=4)

Diet	Initial	Feeding level (FL)				Pooled	*P*-value		
		25%	50%	75%	100%	SEM	Diet	FL	Diet*FL
Moisture ($g.100g^{-1}$)	64.94								
HF		69.0^{b}	68.1^{ab}	67.6^{ab}	66.7^{a}	1.12	N.S	*	*
LF		69.1^{b}	68^{ab}	68.4^{b}	68.8^{b}				
Crude protein ($g.100g^{-1}$)	17.73								
HF		16.8	16.9	16.5	16.9	0.94	N.S	N.S	N.S
LF		16.5	17.1	17.6	17.3				
Fat ($g.100g^{-1}$)	11.63								
HF		8.8^{ab}	10.5^{cd}	11.2^{de}	12.2^{e}	0.81	***	***	***
LF		8.6^{a}	9.9^{bc}	9.8^{bc}	9.4^{abc}				
Ash ($g.100g^{-1}$)	5.27								
HF		6.0	5.5	5.1	4.6	0.49	N.S	***	N.S
LF		5.7	5.3	5.0	5.1				
Energy ($kJ.g^{-1}$)	8.78								
HF		6.9^{ab}	7.5^{bc}	7.8^{cd}	8.2^{d}	0.42	***	***	*
LF		6.5^{a}	7.3^{bc}	7.4^{bc}	7.3^{abc}				

Values are mean (n = 4)

***, $P < 0.001$; *, $P < 0.05$; N.S, non-significantly different (Two-way ANOVA).

[abcdef] For parameters with a significant interaction effect between diet and feeding level. Mean values lacking a common supercript differ significantly (Tukey's HSD; $P < 0.05$).

Supplementary Table S2. Apparent digestibility coefficient (ADC) of dietary nutrient intake in Malabar red Snapper (*L. malabaricus*) for diets HF and LF during the 56-day experimental period (n = 4).

	Diet		SEM	*P*-value
	HF	LF		
Protein (%)	94.1	93.5	0.7	N.S
Fat (%)	98.4	96.6	0.5	*
Carbohydrates (%)	83.5	85.2	1.7	N.S
DM (%)	86.2	85.4	1.4	N.S
NFE (%)	89.1	89.3	2.6	N.S
GE (%)	92.9	92	0.8	N.S
Ash (%)	22.8	13.7	8.4	N.S

Values are mean (n = 4)

***, $P < 0.001$; *, $P < 0.05$; N.S, non-significantly different (T-test).

DM = Dry matter, NFE = Nitrogen free extract, GE = Gross Energy

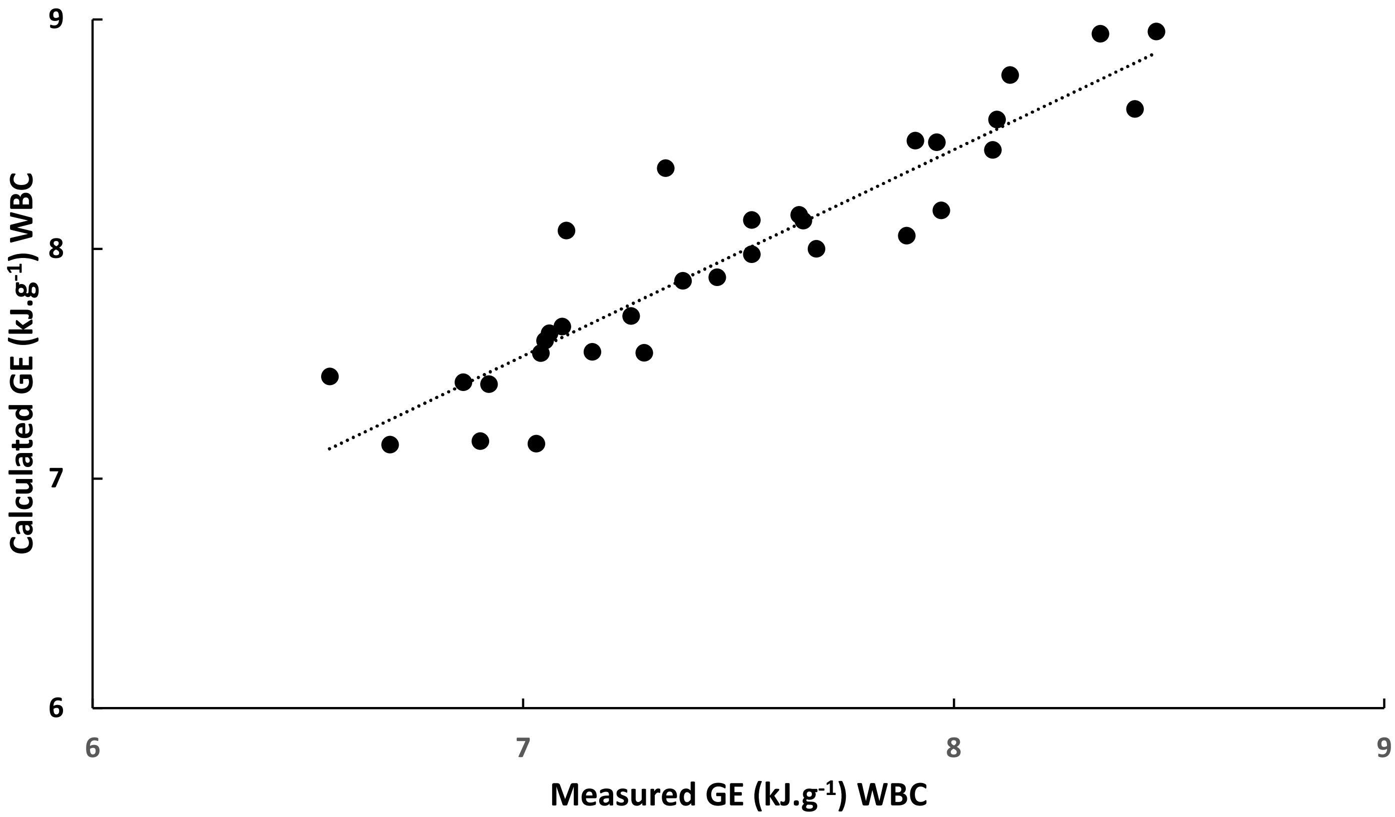

Supplementary Figure 1. Relationship between measured GE ($kJ.g^{-1}$) and calculated GE ($kJ.g^{-1}$) values of whole-body composition (WBC). The calculated GE of WBC were calculated using the mean GE values of Protein Fat and carbohydrate ((23.6, 39.5 and 17.2 $kJ.g^{-1}$, respectively (Blaxter, 1989). The measured GE of WBC were measure using an automated bomb calorimeter (C 5000 Calorimeter, IKA®) under the Isoperibol setting. (R^2 = 0.85).
